# Real-Time Damage Detection in an Airplane Wing During Wind Tunnel Testing Under Realistic Flight Conditions

**DOI:** 10.3390/s25144423

**Published:** 2025-07-16

**Authors:** Yoav Ofir, Uri Ben-Simon, Shay Shoham, Iddo Kressel, Bernardino Galasso, Umberto Mercurio, Antonio Concilio, Gianvito Apuleo, Jonathan Bohbot, Moshe Tur

**Affiliations:** 1 IAI Aviation Group, Ben Gurion International Airport, Lod 7010000, Israel; yoaofir@iai.co.il (Y.O.); ubensimon@iai.co.il (U.B.-S.); sshoam@iai.co.il (S.S.); iddo.kressel@gmail.com (I.K.); 2 CIRA, The Italian Aerospace Research Centre, 81043 Capua, Italy; b.galasso@cira.it (B.G.); u.mercurio@cira.it (U.M.); a.concilio@cira.it (A.C.); 3 Piaggio Aerospace Industries, 17038 Villanova D’Albenga, Italy; gapuleo@piaggioaerospace.it; 4 School of Electrical Engineering, Tel-Aviv University, Tel-Aviv 6997801, Israel; bohbot@mail.tau.ac.il

**Keywords:** structural health monitoring, SHM, damage detection, real-time processing, wind tunnel testing, composite wing, fiber-optic sensing

## Abstract

A real-time structural health monitoring (SHM) system of an airplane composite wing with adjustable damage is reported, where testing under realistic flight conditions is carried out in the controllable and repeatable environment of an industrial wind tunnel. An FBG-based sensing array monitors a debonded region, whose compromised structural strength is regained by a set of lockable fasteners. Damage tunability is achieved by loosening some of or all these fasteners. Real-time analysis of the data collected involves Principal Component Analysis, followed by Hotelling’s *T*-squared and *Q* measures. With previously set criteria, real-time data collection and processing software can declare the structural health status as normal or abnormal. During testing, the system using the *Q* measure successfully identified the initiation of the damage and its extent, while the *T*-squared one returned limited outcomes.

## 1. Introduction

Monitoring the health of structures using technological means (termed SHM for structural health monitoring) has the potential to enhance safety and reduce maintenance expenses by alerting the structure’s operator to perform corrective actions, but only when needed [1,2,3,4,5,6,7,8]. A reliable SHM system may eventually replace schedule-based maintenance with condition-based maintenance [9,10,11,12,13,14], thereby increasing the availability of the structure, airplanes in particular. Among its other missions, detecting damage in a flying structure in *real-time* is a laudable goal of SHM, since it allows proactive response that may save the vehicle and its occupants. Many technologies have been demonstrated for damage detection, including comparative vacuum monitoring (CVM), ultrasonic lamb waves, fiber-optic sensing and more [15,16,17,18,19,20,21,22,23,24,25,26,27,28]. However, achieving *real-time* operation may be hindered by the large amount of data continuously collected by the multiple health-monitoring sensing sites in use. On-board, decision-making processing stands out as a potential viable solution. Examples of already demonstrated algorithms are Kalman filtering [29], wavelet transforms [30], frequency-domain analysis [31] and machine learning techniques (e.g., neural networks and support vector machines (SVMs)) [32]. Principal Component Analysis (PCA) [33,34,35,36,37] does help to reduce the dimensionality of the data but is not a decision-making tool. Indeed, real-time damage detection has been demonstrated using various techniques; however, to the best of our knowledge, it has not been achieved in flight, primarily due to the challenges associated with flying a damaged structure.

This paper replaces actual flights by testing in an industrial wind tunnel, demonstrating *real-time* detection of *adjustable damage* in a composite airplane wing under realistic, potentially envelope-spanning flight conditions. Here, an FBG-based sensing array monitors the flight-induced variable strains in the vicinity of a critical delaminated region of the wing, whose compromised structural strength can be regained by a set of lockable fasteners. The tunability of this originally conceived *adjustable damage* is achieved by loosening some or all of these fasteners. On-board real-time decision-making processing of the collected data starts with the Principal Component Analysis, which highlights the most significant data variations that may indicate structural anomalies. These variations are then quantified by the $T^{2}$ and Q statistics [34,35,36,37], providing one-dimensional measures for identifying deviations from pre-defined normal operating conditions.

It should be emphasized that the main goal of this paper is to bring to the attention of the SHM community the use of wind tunnels for testing SHM methods and algorithms under emulated flight conditions in a repeatable and less expensive way. Employing fiber-optic sensing, as well as the use of the PCA-based damage detection approach, just served as an example. Ultrasonic-based SHM methods and other damage detection SHM algorithms can be equally tested. Also proposed and demonstrated is the idea of adjustable and recoverable damage, which may well help in the testing of different SHM algorithms, easily switching between the ‘healthy’ and ‘damage’ states, including intermediary steps.

This paper is organized as follows: Section 2 and Section 3 present the composite wing and its FBG-based fiber-optic sensing array. The wind tunnel and the data acquisition system are described in Section 4 and Section 5, respectively. Data processing is detailed in Section 6. The results appear and are discussed in Section 7, and a summary and concluding remarks are the subjects of Section 8. Some general considerations related to the use of fiber-optic sensing nets and PCA in SHM are discussed in Appendix A.

## 2. The Composite Airplane Wing with Adjustable Damage

For the SHM system demonstration, a production-standard composite outer-wing segment measuring 2.3 m in length was selected as the test specimen. The damage scenario involved an adjustable debond introduced at a predetermined interface location, to be described below. The growing prevalence of composite structures in aerospace applications stems in part from the manufacturing efficiency and weight savings afforded by adhesive bonding, which increasingly supplants conventional mechanical fastening. Nonetheless, debonding remains a critical failure mode. Its early-stage development perturbs the local strain distribution, making it amenable to early detection via a fiber Bragg grating (FBG) sensor network either surface-mounted on the structure or integrated within it.

**Figure 1:**
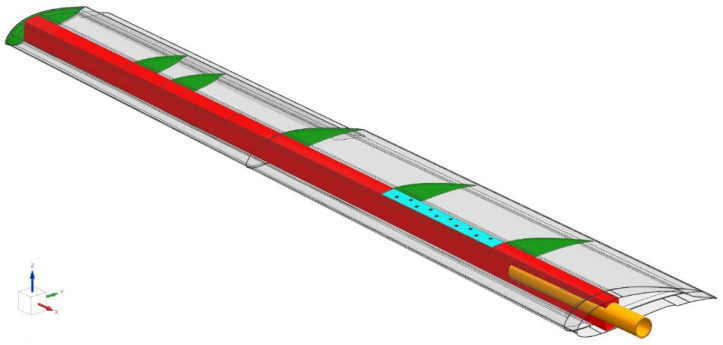
The composite wing: inner structure.

**Figure 2:**
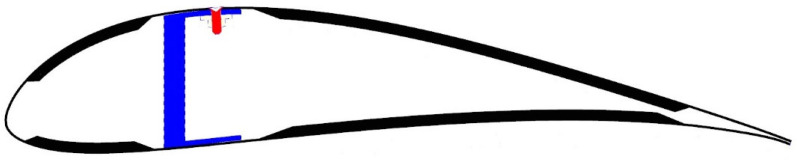
A cross-section of the composite wing, showing the spar, the skin and one of the holes, occupied by a fastener and its under-skin nut.

**Figure 3:**
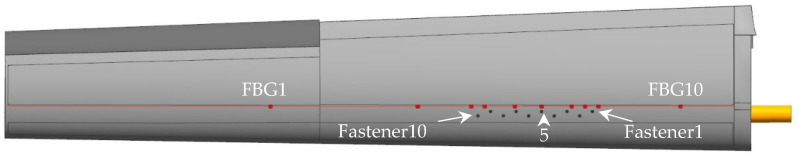
An engineering drawing of the skinned wing, showing the 10 fasteners and 10 FBGs.

**Figure 4:**
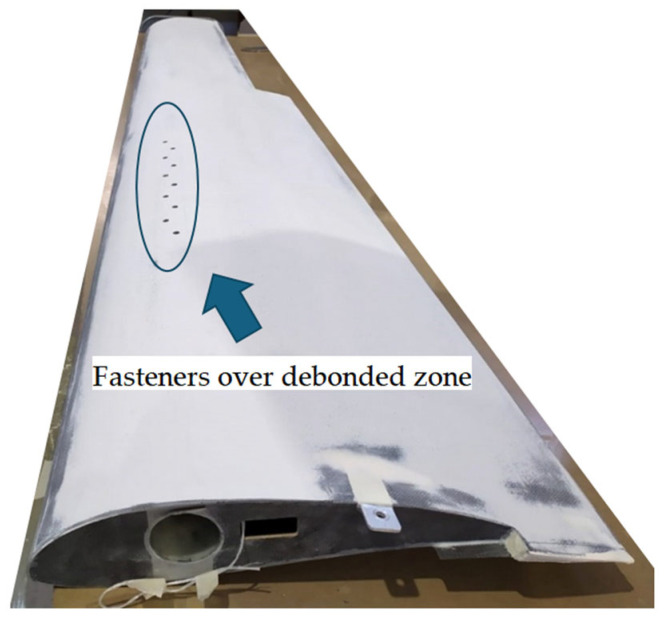
The wing. The arrow points to the fasteners over debonded zone (aileron not shown).

The wing, Figure 1, is a bonded assembly comprising a ‘C’-section front spar, ribs, fuselage fitting and skins. The ribs, bonded to the spar, also support the aileron fittings and an electrical actuator. The lower and upper skins are mostly made of a foam core sandwich, apart from their interfacing area with the spar, where the foam core is replaced by additional composite plies, as shown in Figure 2. The skins are bonded to the spar and the ribs without any fasteners, except for the second bay, where no adhesive was applied between the upper skin and the spar, forming a 42.5 cm long debond. Instead of the adhesive, ten firmly tightened fasteners were used to replace the adhesive, thereby restoring the structural integrity of the wing. Through the use of this approach, the partial removal of fasteners creates structural damage, whose magnitude depends on the number and choice of removed fasteners. However, to positively eliminate uncontrolled damage propagation into adjacent bays, the two outermost fasteners (Nos. 1 and 10 in Figure 3) were designed to serve as a damage-arresting feature. This concept of adjustable damage was first statically tested to verify a safe flight envelope. The maximum operational wing limit load was defined as 113 kg (1110 N), enabling maneuvers at high *g* loads. Figure 4 shows a photo of the wing.

## 3. The Fiber Sensing Array and Data Acquisition System

**Figure 5:**
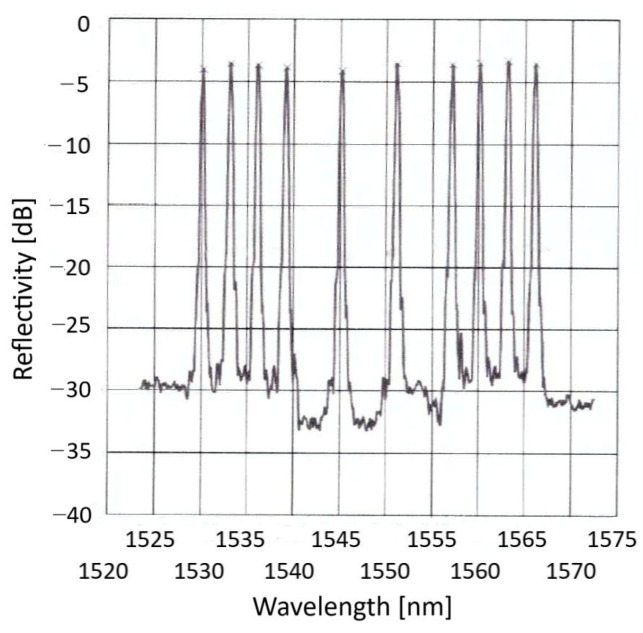
The reflection spectra of the 10 FBGs.

A polyimide-coated (*ϕ*145 μm) optical fiber sensing array, having 10 fiber Bragg gratings (FBGs) inscribed along its length, was placed parallel to the fastener line and securely attached to the skin surface using a structural adhesive, as shown in Figure 3 (this work, boasting real-time operation, could not use distributed fiber-optic sensing (DFOS), mainly due to the large amount of data generated over meters of fiber at the required sampling speed). The spectra of the array of FBGs, Figure 5, indicate quite uniform reflectivities of more than 35% at 1530, 1533, 1536, 1539, 1545, 1551, 1557, 1560, 1563 and 1566 nm (within −0 + 0.06 nm). Bandwidths at −3 dB were 0.383 ± 0.008 nm, and sideload suppression was better than 15 dB. The fiber array was glued to the thoroughly cleaned wing surface and then mechanically protected by a cured 3 cm wide and 0.1 mm thick layer of epoxy resin-impregnated glass fiber fabric. Seven FBGs were spaced along the damage zone and three outside, for reference. A well-secured egress point leads a 3 mm fiber cable through the wind tunnel floor to a commercial dynamic FBG interrogator.

**Figure 6:**
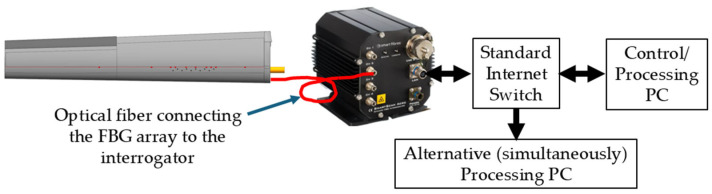
The data acquisition setup included the FBG interrogator, an internet switch, a Control and Processing PC and another computer that simultaneously received the FBG data broadcast by the interrogator, allowing it to process the data in ways different from those of the other computer (not used in this work).

The data acquisition system, Figure 6, was based on Smart Fibres Ltd. The SmartFibre Aero interrogator is capable of simultaneously reading four independent FBG arrays, with more than 10 FBGs per array. It uses a tunable laser, whose output, covering 40 nm in 0.1 nm steps, is split among the four arrays. For each independently detected array, the scan of the laser generates a wavelength-tagged temporal trace of the light intensity which returns from the FBGs of the array, effectively constituting a discrete spectrum of its FBGs. Identifying intensity values that exceed an adjustable threshold, the included electronic hardware defines the wavelength of peak reflection, $\lambda_{Bragg}$, of each FBG as the spectral center of gravity of its measured spectrum. Scanning its full spectrum at speeds up to 2500 times per second, the collected wavelength values of the peaks of all involved FBGs are *continuously* sent to the Control/Processing PC in a binary format via an internet connection (UDP/IP for data and TCP/IP for configuration). In order not to miss some unexpected phenomena, all experiments reported below used a relatively high sampling (scanning) speed of 1250 readings per second. In retrospect (see Section 6), the key dynamics were significantly slower, and data processing was conducted on time-decimated data.

Rather than connecting the Control/Processing PC directly to the interrogator, an internet switch was added, and the interrogator was configured to broadcast its data to another local or even remote computer for simultaneous processing using different algorithms.

Real-time SHM operation, being one of the core goals of this work, is understood here, in the context of SHM and damage monitoring of flying structures, as the ability of the system to provide health assessments of the structure at a user-defined frequency. The PCA-based SHM described in this paper, capable of generating graphical assessments of the wing ‘health’ at sub-minute intervals, was implemented in the following manner: (*i*) after being properly configured, the Control/Processing PC was programmed to continuously receive the interrogator data, converting the incoming binary representations of the measured data to decimal wavelengths and storing the results on its disk; (*ii*) every T s (e.g., a few tens, tested down to 10 s) a T-long segment of data was read from the disk and quickly processed by the PCA algorithm (the matrix–vector multiplication of Equation (3)), displaying the resulting $T^{2}$ and Q measures on the computer screen, together with appropriate, engineering-motivated thresholds. At such a monitoring rate, even the initiation of damage could be detected in time and disaster-preventing actions could hopefully be taken.

## 4. The Wind Tunnel Setup

**Figure 7:**
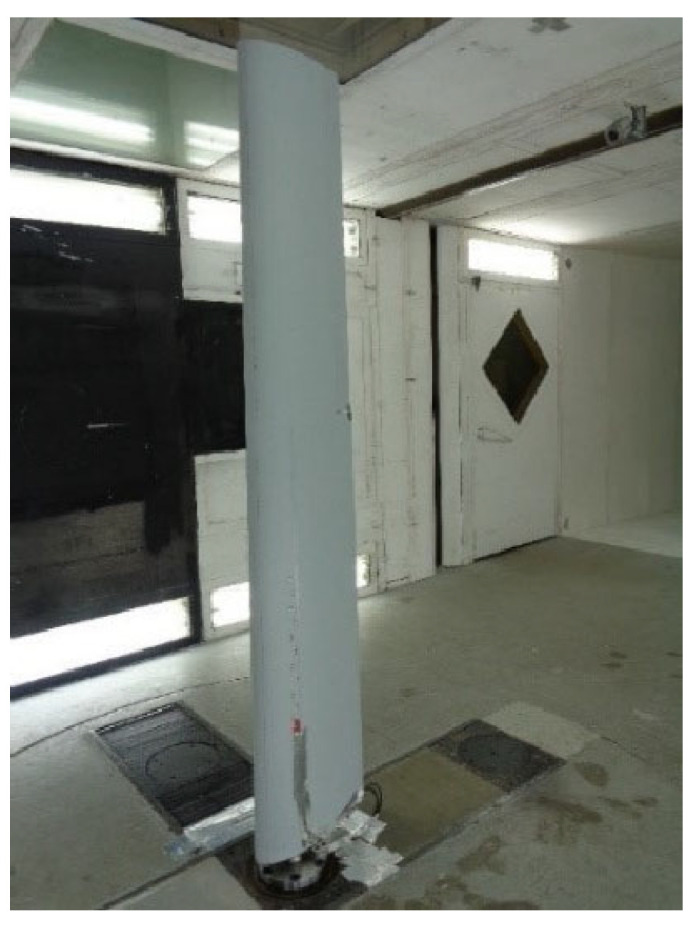
The composite wing mounted on a rotatable model support of the wind tunnel. Airflow arrives from the left.

The experiment was executed in the IAI subsonic wind tunnel. The wing was firmly mounted on the rotatable tunnel model support, Figure 7, equipped with a balance to measure the aerodynamic forces and moments experienced by the wing during testing. Health monitoring of the wing under variable wind speeds and fastener settings was performed using the data acquisition and health measurement system. The interrogator, switch and Control/Processing computer were all located in the tunnel control room.

## 5. Data Processing Methodology, Both Preparatory and Real-Time

In this work, real-time processing of continuously streaming data segments is based on processing measurements, taken for the *current* state of the wing against a previously established mathematical model, calculated for the ‘healthy’ wing (or another ‘reference state’ of choice). Following [33,34,35,36,37], we enlisted the Principal Component Analysis algorithm to reduce the dimensionality of the data, thereby creating an efficient computational model that allows *live* processing of the incoming in-flight data. Tools borrowed from multivariate data analysis were then used to report real-time deviations from the reference (e.g., ‘healthy’) state. The subsections below detail the employed procedures.

### 5.1. Principal Component Analysis

Given a complex, multivariate data set (i.e., continuously streaming segments of FBG readings from all FBG sensors), the PCA algorithm transforms the original data into a new set, consisting of uncorrelated features that reveal the dynamics of importance and potentially used to *discard* redundancy and noise, providing significant data reduction [34,35,36,37].

First, an incoming segment (say, 10 s long) of the *reference* data set is organized as an n×m matrix, X, where, in our case, the number of columns, m, is the number of FBGs (representing 10 measured variables), and the number of rows, n, is the number of observations (time samples collected during a particular experiment). The data in each *column* is optionally frequency-filtered and/or decimated and then normalized to zero mean and unity variance, resulting in the matrix $X_{norm}$. Based on the given normalized data set, the PCA algorithm aims to find m orthogonal *directions* in the m vector space, such that the projection of the raw data in these directions will show maximum variance for one direction and decreasing variances for the others. It turns out that these directions and their associated variances are the eigenvectors and eigenvalues of the m×m covariance matrix, $C_{X}=X_{norm}^{T}\cdot X_{norm}/\left(n-1\right)=P\Lambda P^{T}$. After proper sorting (if required), Λ is a diagonal matrix, whose diagonal holds the ordered eigenvalues, $\lambda_{1}\geq \lambda_{2}\geq ,\ldots ,\geq \lambda_{m}$ (all ≥0 since $C_{X}$ is non-negative definite). The columns of the orthogonal m×m matrix, P, are the orthonormal eigenvectors of $C_{X}$, which are also the sought-after *directions* in m space. The normalized input data, $X_{norm}$, can now be transformed into another n×m matrix, T, using $T=X_{norm}P$. For a given measurement, say, $\left\{X_{norm,i_{0,}j},j=1,\ldots ,m\right\}$, the corresponding elements of T, $\left\{T_{i_{0}j},j=1,\ldots ,m\right\}$, are the *principal components* of that measurement, also called the *scores*. Interestingly [33], the covariance matrix of T is simply the diagonal matrix Λ, so that all columns of T are uncorrelated and their variances are the corresponding elements of Λ.

PCA is highly effective in those cases where most of the eigenvalues are very small, and only a few, say, 1,…,l≪m, are considerably larger. Significant data reduction can be achieved by judiciously selecting in P only the eigenvectors which correspond to the highest l eigenvalues. Mathematically, let $P_{l}$ contain the first l columns of P. Then, the reduced data representation becomes

(1) $$T_{l}=X_{norm}P_{l},$$

where the reduced nxl score matrix $T_{l}$ has now only l columns. It is common [33,34,35,36,37] to approximate that part of $X_{norm}$ which is covered by the reduced $T_{l}$ by ${\hat{X}\equiv T}_{l}P_{l}^{T}=X_{norm}P_{l}P_{l}^{T}$ ($P_{l}$ is not orthogonal), so that $X_{norm}$ can be expressed as the sum of the reduced part, $\hat{X},$ and a *residual*, R (I is the m×m identity matrix):

(2) $$X_{norm}=\hat{X}+(X_{norm}-\hat{X})\equiv \hat{X}+R\to R=X_{norm}(I-P_{l}P_{l}^{T})$$

The adequacy of the choice of l can be roughly estimated by the ratio of the sum of variances in the l columns of $T_{l}$ over the sum of all variances—$\sum_{1}^{l}\lambda_{j}/\sum_{1}^{m}\lambda_{j}$—the closer to unity, the better the expected accuracy of the reduced representation.

In many cases, the l chosen principal components reveal the most dominant trends of the data and contain most of the information of interest, covering most of the variance in the data.

In summary, our damage detection data analysis proceeds along the following sequential steps: (*i*) A reference state of the structure is selected. (*ii*) A PCA model (normally, reduced) is then generated. (*iii*) The number of principal components to be used, l, is judiciously selected, thereby defining the $P_{l}$ matrix and the reference scores, $T_{l}^{ref}$. (*iv*) New measurements, taken under modified conditions of the structure (e.g., damaged or under different flying conditions), $X^{new}$(of dimensions $n^{\prime }\times m$, where $n^{\prime }$ may be different from n), are normalized using the *means and standard deviations of the reference data*, resulting in $X_{norm}^{new}$. (*v*) New scores, $T_{l}^{new}$, are then computed by applying Equation (1) to the new normalized data:

(3) $$T_{l}^{new}=X_{norm}^{new}P_{l}$$

(*vi*) Finally, $T_{l}^{new}$(column vector, $\left\{t_{i}^{new},i=1,\ldots ,n\right\}$), together with the PCA model’s Λ and $P_{l}$, are used by the measures described below to determine deviations, the significance of which is to be evaluated by limits set by the *user*.

### 5.2. Hotelling $T^{2}$ Measure

For each new measurement of the m variables, the value of the $T^{2}$ measure is given by

(4) $$T_{i}^{2}\equiv t_{i}\Lambda_{l}^{-1}t_{i}^{T}=x_{i}P_{l}\Lambda^{-1}P_{l}^{T}x_{i}^{T}$$

Here, x represents a *row* of m measured values, while t stands for their corresponding row of l<m principal components. $\Lambda_{l}$ is the top-upper-left part of Λ, containing only the largest l values, and the matrix $P_{l}$ was introduced in Section 5.1, calculated from similar measurements of the *reference* state.

It has been shown in *statistics* [33] that $T^{2}\left(x\right)=Constant$ defines an l-dimensional ellipsoid in m-dimensional space, whose surface and inner volume contain all measurement points for which the probability of deviating from the zero-centered mean is lower than a value related to the Constant through the Hotelling T-square statistic [33]. Measurements resulting in points outside the ellipsoid represent *deviations*.

Here, *within the context of the reduced PCA model*, a new (normalized) measurement $x_{i}$ (or its $t_{i}$ counterpart), taken under structural conditions different from those under which the reference PCA model was established (e.g., in the presence of damage or more extreme operational conditions), is then plugged into the right-hand side of Equation (4). If the result exceeds a user-defined value, it is then flagged as a deviation requiring attention.

### 5.3. The Q Measure

But what if too few principal components are retained, resulting in a non-negligible residual $R=X_{norm}-\hat{X}$ of Equation (2) For a given new measurement, comprising the 1xm-dimensional vector $x_{i}$, a common way [33,34,35,36,37] to address this question is to calculate the Q measure given by (${\hat{x}}_{i}=x_{i}P_{l}P_{l}^{T}$, Section 5.1)

(5) $$Q_{i}={\left(x_{i}-{\hat{x}}_{i})\cdot (x_{i}-{\hat{x}}_{i}\right)}^{T}=x_{i}\left(I-P_{l}P_{l}^{T}\right)x_{i}^{T}.$$

Thus, $Q_{i}$ represents the sum of squares of the distance of $x_{i}-{\hat{x}}_{i}$ from the l-dimensional space that the reduced PCA model defines. It can be shown [33] that $Q_{i}$ can also be expressed as $Q_{i}=\sum_{j=l+1}^{m}t_{j}^{2}$, thereby representing the residual subspace spanned by the vectors that were not included in the reduced model. It is generally more sensitive than the $T^{2}$ statistic [33], but more prone to noise.

Large deviations in Q values from those obtained for the reference case should be addressed as warnings.

## 6. Test Procedure

Overall, the entire test procedure involved a few wind tunnel runs, each lasting 12–22 min, Table 1. The wing aileron was set to zero position by its actuator. Healthy wing tests were performed with tightened fasteners. Different degrees of damage were emulated by opening either 5 fasteners (B1, B3, B5, B7, B9; Figure 3) or all 10 fasteners. Here, we report a few example cases with and without damage.

**Figure 8:**
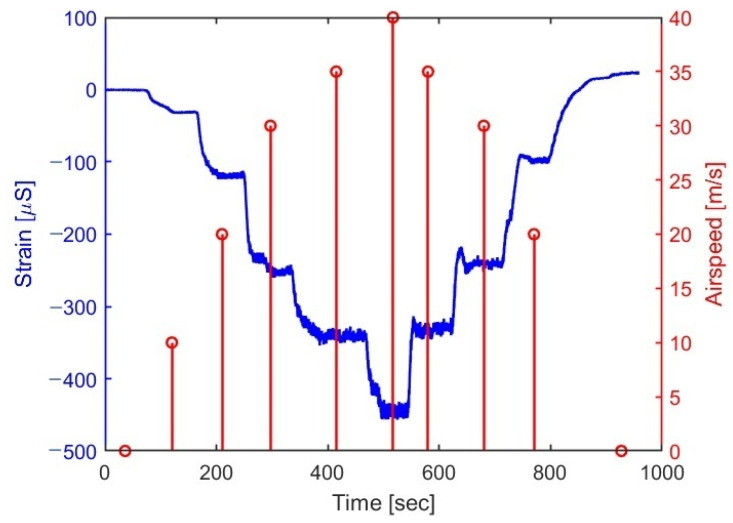
Typical strain (left axis) of FBG 8 (Figure 3) against time in a typical tunnel run, together with the recorded airspeed (stem plot, right axis).

Each experiment started by setting the strains of all FBGs to zero. Figure 8 shows the strain in a typical tunnel run, together with the airspeed at the center of each step. At an attack angle of 4 degrees, all FBGs were under compression during the run. Note, though, the *positive* strain readings at the end of the run (~24 μS), indicating a temperature effect. Indeed, in this experiment, the temperature started at 30° Celsius, increasing to 35.7° at the maximum airspeed of 41.8 m/s, ending at 34°. Assuming the wing to straighten up elastically at the end of the run to its run-start position, we can roughly estimate the dependence of the FBG readings on temperature as 6 μS/°C (=24/4), close enough to the known values for FBGs glued to the surface of composites [38,39].

**Figure 9:**
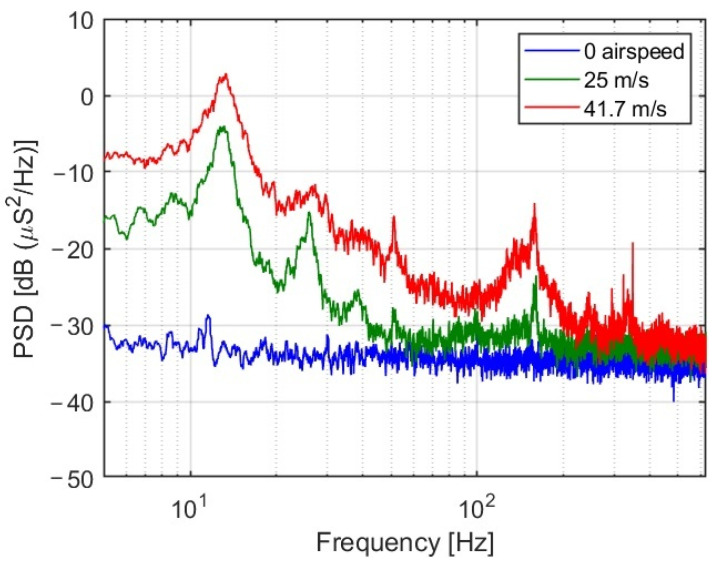
Power spectral densities of the strain readings of FBG 8 for a healthy wing, Case 1, calculated from segments of constant airspeed at zero airspeed (bottom, blue), 25 m/s (middle, green) and 41.7 m/s (top, red), all taken at 1250 samples/s. Note the weaker harmonics, clearly visible in the middle, green trace. The sources of the significantly weaker events at frequencies above approximately 100 Hz have yet to be identified.

The uncertainty of the strain measurements, directly estimated from the zero-airspeed portions of the trace, is less than 1 μS (std) in all experiments. Another common feature of all traces is the observed increase in thickness with the tunnel airspeed. Rather than representing noise, analysis in both time and frequency domains reveals a lift-force-dependent *vibrational* mode of the wing at 13 Hz, as shown in Figure 9.

Since these spectra are shared by all runs (at the quoted airspeeds), healthy and damaged alike (up to 1–2 dB), and since the strength of the 13 Hz fundamental mode clearly dominates the dynamics of the events, it appears that all experiments could have been sampled at 125 Hz, rather than 1250 Hz, considerably reducing the amount of stored and processed data.

The following section focuses on attempts to apply the $T^{2}$ and Q tests to both run segments of equal airspeed (Section 7.1) and to the complete runs (Section 7.2).

## 7. Results and Their Analysis

### 7.1. Comparing Segments of Equal Airspeed

**Figure 10:**
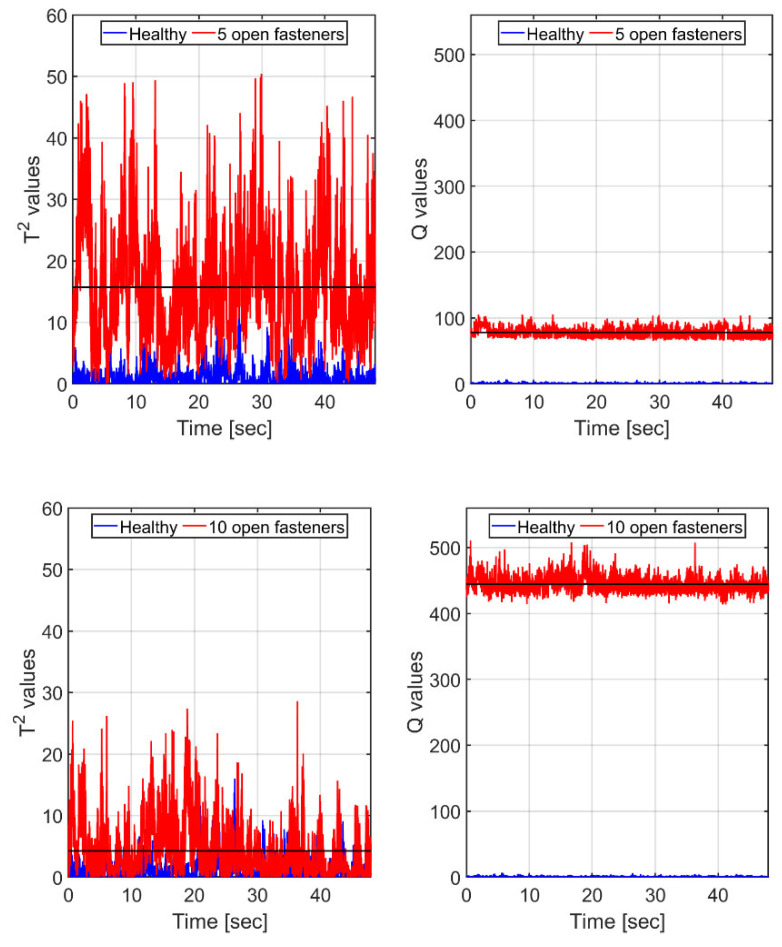
The equal-speed scenario: $T^{2}$ and Q values (red curves) for the wing, with a ‘damage’ of 5 (**top** frames) and 10 fasteners (**bottom** frames). Black horizontal lines denote the corresponding means of the strain. Results are based on *single* principal component scores. Blue (**bottom** curves): corresponding $T^{2}$ and Q values, calculated for the *healthy* case, are based on its *own* PCA model.

With real-time SHM damage detection applications in mind, a possible scenario may involve continuous (or intermittent) comparisons of segments of current readings of the sensing net with stored values of the healthy states of the vehicle under the same flight conditions. We thus isolated segments of equal speed of approximately equal durations and subjected the strain traces (compensated for small differences in temperature and airspeed) to $T^{2}$ and Q tests. Case 1 (serving as the healthy/reference state) and its 10 FBGs’ readings in the segment of max speed (41.8 m/s) were used to establish the reference PCA model, following the procedure outlined in Section 5.1. Since the largest eigenvalue of the covariance matrix represented 91% of the total variance, scores based on the leading principal component were subjected to $T^{2}$ and Q analyses, resulting in Figure 10 (5 fasteners open, Case 2) and 8 (10 fasteners open, Case 3). Note that in each case we also show (bottom, blue) the $T^{2}$ and Q values of the healthy case against itself.

It is clearly seen in Figure 10 that for scores based on a *single* principal component of the healthy state, the Q measure proves itself to be very sensitive to the presence of the ‘damage’, dramatically increasing in value with its growing extent. In contrast, the $T^{2}$ one is much noisier, having a mean value for the more ‘damaged’, 10-open-fastener case *lower* than the mean for the less ‘damaged’, 5-open-fastener one.

**Figure 11:**
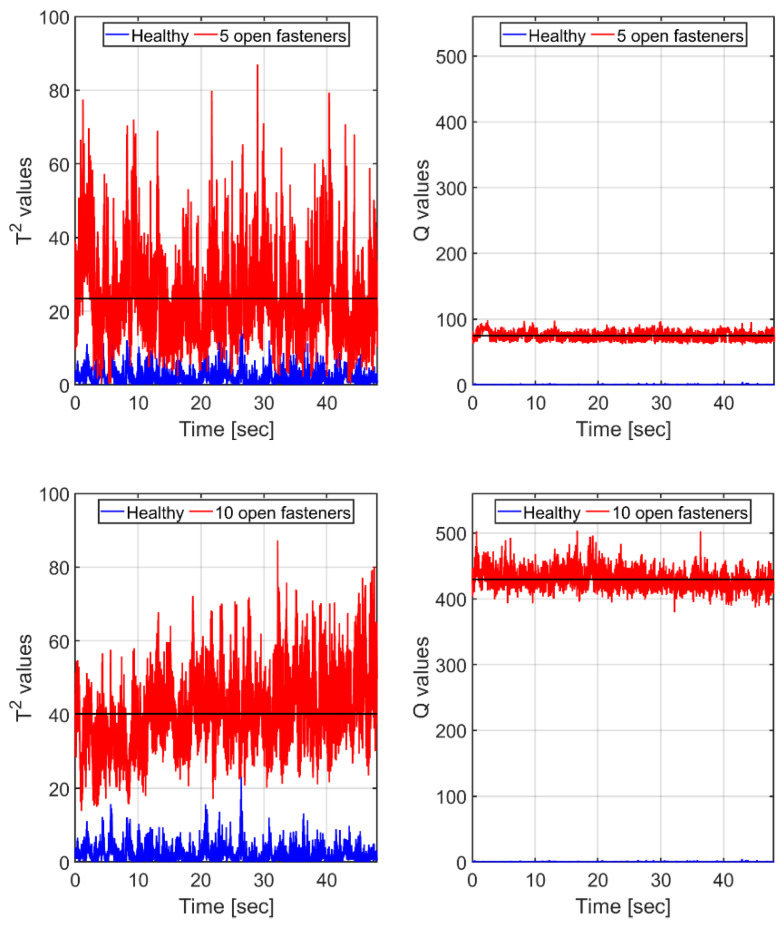
The equal-speed scenario: same as Figure 10, but here, scores calculations are based on the *two* leading principal components of the healthy model. $T^{2}$ and Q values (red curves) for the wing, with a ‘damage’ of 5 (**top** frames) and 10 fasteners (**bottom** frames). Black horizontal lines denote the corresponding means. Blue (**bottom** curves): corresponding $T^{2}$ and Q values, calculated for the *healthy* case, based on its *own* PCA model.

Recalling that the first principal component, on which Figure 10 is based, represents only 91% of the variance in the healthy model, we recalculated the curves of Figure 10 using scores containing two leading principal components, as shown in Figure 11, now covering 95.2% of the variance.

With two principal components, both $T^{2}$ and Q means show higher values for the more damaged state, while still, the mean-to-std ratio is much higher for the Q measure. Note that the values of $T^{2}$ increase while those of Q decrease. This trend continues as increasingly more principal components are added.

### 7.2. Comparing Complete Traces

In this section damage is assessed by applying $T^{2}$ and Q to complete runs (mimicking take-off, cruise and landing conditions). In these runs, the airspeed starts at zero, increases in steps to a maximum value (~40 m/s) and then decreases, also in steps, back to rest; see Figure 5, which portrays the induced strain in one of the FBGs during a run. No effort was made to fully synchronize the temporal profile of the airspeed to be the same for all runs. Again, Case 1 served as the healthy case, with the largest eigenvalue of its complete-trace-based covariance matrix representing 99.9% of the total variance. Cases 2 and 3 represented the initial and final stages of the damage, respectively.

**Figure 12:**
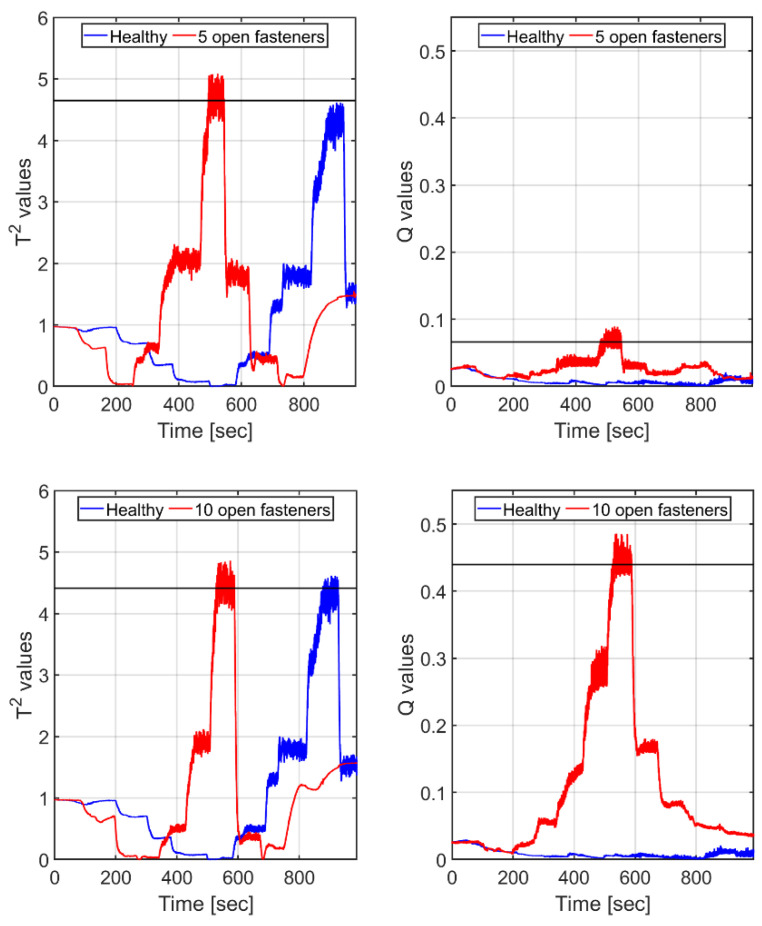
Whole-trip scenario: $T^{2}$ and Q values (red curves) for the wing, with a ‘damage’ of 5 (**top** frames) and 10 fasteners (**bottom** frames). Black horizontal lines denote the corresponding means, computed from the upper segments of the red curves. Results are based on *single* principal component scores. Blue (**bottom** curves): corresponding $T^{2}$ and Q values, calculated for the *healthy* case, based on its *own* PCA model.

Figure 12 shows the $T^{2}$ and Q measures for this whole-trip scenario, together with their means at their highest values, which occur during the period of maximum airspeed.

Comparing the airspeed temporal profiles of Cases 2 and 3 with the $T^{2}$ and Q curves of Figure 12, we found that the highest values of both $T^{2}$ and Q**,** used for the indication of damage, temporally align with the moments when the maximum airspeed occurs. Since the two runs were not temporally identical, there is a slight shift in the regions of maximum value between the top and bottom panes in the figure.

Clearly, the Q measure detects the damage, outputting a mean value that is not only much higher than its corresponding one but also increases with the extent of the damage. As for the $T^{2}$ measure, its high mean values for the two types of damage (4.6 for the 5-open-fastener case and 4.4 for the 10-open-fastener one) are not commensurate with the extent of the corresponding damage, and the difference between the two values is minor. Also note that the $T^{2}$ values for the healthy case against its own PCA model are as large as for the damage case (note that the figure shows the results for Cases 2 and 3 in full but truncates those for Case 1, whose run was a bit longer, yet the PCA model was based on the whole run of healthy Case 1).

## 8. Summary and Conclusions

This study demonstrates the use of wind tunnel experiments to test the SHM capabilities of a damage detection approach that allows for real-time assessment of damage in an aircraft wing located inside the tunnel. The proposed approach starts with the establishment of a PCA model for the healthy state of the wing using strain readings from a fiber-optic sensing net during emulated tunnel flights. The damage is a debond region of the wing, repaired back to its healthy state by 10 tightened fasteners. Through the use of the preprepared healthy PCA model, repeated strain measurements of the wing by the same sensor net in the presence of damage (selected fasteners are opened to create damage of varying extent) are then quickly converted in real-time to scores, whenever possible using only the first one or two leading principal components, and processed to produce $T^{2}$ and Q measures, which are intended to indicate the presence and extent of damage.

Comparing readings of healthy and damage states of the structure during constant airspeed conditions, we found the Q measure to generate values that not only were quite distinct from those of the healthy state but also monotonically grew with the extent of the damage. This was not the case for the $T^{2}$ measure. This conclusion remained valid when complete trips were compared.

Clearly, engineers are the ones who interpret specific SHM numerical outputs as damage of a given severity, using thresholds grounded in engineering experience and judgment. This step was avoided in this study, at least until more experience is gained with our proposed and demonstrated approach.

Note that such an SHM system can be easily integrated into modern aircraft avionics, especially in cases where internet communication is supported. Some commercially available interrogators have successfully passed thorough environmental tests, including RFI/EMI, demonstrating their compatibility with aircraft avionics. As for power consumption, modern aerospace-designed interrogators require no more than a few tens of Watts. Finally, most modern aircraft have available computing power that can easily perform the matrix–vector multiplication required by the PCA algorithm once a PCA model has been already established. Telemetry can then provide transmission to ground of either the raw PCA results or only those values exceeding pre-designed thresholds.

Wind tunnel experiments may offer a more cost-effective and technically convenient means of validating both old and new SHM approaches compared to actual flights. They also offer a more controlled and repeatable environment. More wind tunnel studies, involving genuinely damaged structures, combined with state-of-the-art real-time health monitoring algorithms (including those based on machine learning and AI) have the potential to accelerate the industrial adoption of SHM in aeronautics.

## Figures and Tables

**Table 1:** Reported cases covering different airspeeds and damage conditions.

Experiment	Airspeed [m/s]	Max Lift Force [kgf]	Angle of Attack [°]	Number of Removed Fasteners
1	0–41.7	108.5	4	0
2	0–41.8	108.5	4	5
3	0–42	107.8	4	10

## Data Availability

Data will be provided under specific conditions and if necessary.

## Appendix A

### Appendix A.1. On the Use of Fiber Sensing Networks in SHM Applications

- The proper operation of the FBG fiber sensing net requires the correct placement of each FBG to ensure linear strain transfer between the FBG and the underlying measured structure.
- Optical fiber integration in aircraft structures is not limited by length or size. Sensor integration, including embedding, surface bonding using a dedicated sensing mat, egress/ingress concepts and repairability were demonstrated in the EU SARISTU project [40] on a real aircraft structure demonstrator. Yet practical issues, such as price, maintenance and the inability of the FBG to sense remote events that do not affect the strain surrounding the FBG, may limit the use of FBG-based sensing techniques to monitor only discrete troublesome hot spots.
- Optical fibers, including FBGs inscribed within them, are well known for their ability to withstand harsh environmental conditions [41,42]. This robustness makes them ideal for applications in communications, aerospace, civil engineering, oil and gas and structural health monitoring. However, besides being sensitive to strain, the peak reflection wavelength ($\lambda_{B}$) of the FBG is also influenced by temperature (through its effect on fiber core, cladding and coating [38]) and humidity (coating only [39,43]). A robust and commonly preferred method to eliminate the temperature sensitivity of the strain-measuring FBG array is to attach or embed an auxiliary strain-isolated, temperature-only sensing FBG array quite close to the primary array. Once the temperature sensitivity of the primary array is accurately characterized, the data from the other array can be used to extract temperature-independent strain information from the primary FBG array. As for humidity, compensation can be implemented provided that the actual value of humidity in the vicinity of the FBG is measured independently and the dependence of $\lambda_{B}$ on this parameter has been calibrated.
- FBG sensing for SHM is commonly used for strain monitoring. Thus, buckling, delamination, debonding and cracks, all types of damage associated with a loss of stiffness, can be handled by the proposed system after thorough testing and verification, preferably combined with a digital twin.
- Long-term operation of such a damage-detecting SHM system depends on quite a few factors. Modern FBG interrogators have reasonable long-term stability and can be easily calibrated, if necessary. While the FBGs themselves are known to be quite stable, their actual behavior also critically depends on the stability of the matrix they are embedded in or attached to. Under these circumstances, timely calibration will be required. The dynamic effects of temperature and humidity will have to be compensated for, as described above. In any case, it is too early to obtain a reliable estimate of the expected measurement error of such a system over an extended period.

### Appendix A.2. On the Use of PCA-Based Algorithms

- Despite the high degree of similarity among a family of same-model structures, a PCA model constructed for one family member may be used for another member only after thorough verification and validation.
- Under realistic flight conditions, involving mixed-mode loading and a noisy environment, a single statistic may not be sufficient to definitively assess the health status of the structure.
- The operation of the PCA-based algorithm must be tested under dynamic nonlinear maneuvers of the structure.
